# Supplementary material for: Machine Learning Accelerates Crystallization for Structure Determination
Source: Angew Chem Int Ed Engl. 2026 May 4;65(25):e1218503. doi: 10.1002/anie.1218503 (PMC13266958; doi:10.1002/anie.1218503)

checkCIF/PLATON report

Structure factors have been supplied for datablock(s) lcz071

THIS REPORT IS FOR GUIDANCE ONLY. IF USED AS PART OF A REVIEW PROCEDURE FOR PUBLICATION, IT SHOULD NOT REPLACE THE EXPERTISE OF AN EXPERIENCED CRYSTALLOGRAPHIC REFEREE.

No syntax errors found. CIF dictionary Interpreting this report

Datablock: lcz071

Bond precision:	C-C = 0.0201 A		Wavelength=1.54184
Cell:	a=15.5671(2)	b=13.1301(2)	c=16.7111(3)
	alpha=90	beta=91.458(1)	gamma=90
Temperature:	100 K		

	Calculated	Reported
Volume	3414.60(9)	3414.60(9)
Space group	P 21/c	P 1 21/c 1
Hall group	-P 2ybc	-P 2ybc
Moiety formula	C29 H10 Ag3 Cl F18 N6 O2 [+ solvent]	C15 H3 Ag3 F18 N6, C14 H7 Cl O2
Sum formula	C29 H10 Ag3 Cl F18 N6 O2 [+ solvent]	C29 H10 Ag3 Cl F18 N6 O2
Mr	1175.49	1175.49
Dx,g cm-3	2.287	2.287
Z	4	4
Mu (mm-1)	15.705	15.705
F000	2248.0	2248.0
F000'	2260.00	
h,k,lmax	19,16,21	19,16,20
Nref	7335	6975
Tmin,Tmax	0.154,0.111	0.410,1.000
Tmin'	0.043	

Correction method= # Reported T Limits: Tmin=0.410 Tmax=1.000
AbsCorr = MULTI-SCAN

Data completeness= 0.951 Theta(max)= 78.403

R(reflections)= 0.0891(4764) wR2(reflections)= 0.2243(6975)

S = 1.115 Npar= 529

The following ALERTS were generated. Each ALERT has the format
test-name_ALERT_alert-type_alert-level.
Click on the hyperlinks for more details of the test.

Alert level B

PLAT110_ALERT_2_B ADDSYM Detects Potential Lattice Translation ... ? Check

Author Response: The host has higher symmetry, but the co-crystallized molecules prevent the possible additional symmetry. The space group was checked with Platon which indicated that there was pseudo-translation present but that the correct P 21/c.

PLAT112_ALERT_2_B ADDSYM Detects New (Pseudo) Symm. Elem I 100 %Fit

Author Response: The host has higher symmetry, but the co-crystallized molecules prevent the possible additional symmetry. The space group was checked with Platon which indicated that there was pseudo-translation present but that the correct P 21/c.

PLAT113_ALERT_2_B ADDSYM Suggests Possible Pseudo/New Space-group I2/a Check
WARNING: Disordered Atoms Excluded from Analysis
Check Model Parameter Symmetry for Reflection Data Support

Author Response: The host has higher symmetry, but the co-crystallized molecules prevent the possible additional symmetry. The space group was checked with Platon which indicated that there was pseudo-translation present but that the correct P 21/c.

PLAT342_ALERT_3_B Low Bond Precision on C-C Bonds .... 0.02011 Ang.

Author Response: Disordered structure.

Alert level C

PLAT213_ALERT_2_C Atom F00X has ADP max/min Ratio ..... 3.1 prolat

```
PLAT234_ALERT_4_C Large Hirshfeld Difference Ag01     --N00E     .       0.17 Ang.
PLAT234_ALERT_4_C Large Hirshfeld Difference Cl00     --C01L     .       0.17 Ang.
PLAT241_ALERT_2_C High   'MainMol' Ueq as Compared to Neighbors of       O00P Check
PLAT242_ALERT_2_C Low    'MainMol' Ueq as Compared to Neighbors of       Ag01 Check
PLAT906_ALERT_3_C Large K Value in the Analysis of Variance ......     19.946 Check
PLAT906_ALERT_3_C Large K Value in the Analysis of Variance ......      5.532 Check
PLAT906_ALERT_3_C Large K Value in the Analysis of Variance ......      2.856 Check
PLAT911_ALERT_3_C Missing FCF Refl Between Thmin & STh/L=    0.600         47 Report
                 1  1  0,   9  0  0,  11  4  0,  -9  0  2,   0  0  2,   2  0  2,
                 3  8  2,  -3  7  3, -14 10  4,  -5  6  4,  -3  6  4,  -2  7  4,
                 2  0  4,   2 15  4,   9  0  4,  -3  7  5,  15  8  5,  -1  4  6,
                 4  5  6,  -1  5  7,   6  1  7,   6  2  7,  14  8  7,  -1  0  8,
                14  8  8,   2  5 10,   3  6 10,   1 13 11,   2 13 11,   4  6 11,
                     (   17 More Missing: see the .ckf listing file)
PLAT934_ALERT_3_C Number of (Iobs-Icalc)/Sigma(W) > 10 Outliers ..          1 Check
                 5  5 17,
PLAT971_ALERT_2_C Check Calcd Resid. Dens.  0.81Ang From Ag02            2.14 eA-3
PLAT971_ALERT_2_C Check Calcd Resid. Dens.  0.85Ang From Ag01            2.09 eA-3
PLAT971_ALERT_2_C Check Calcd Resid. Dens.  0.85Ang From Ag03            2.01 eA-3
PLAT971_ALERT_2_C Check Calcd Resid. Dens.  0.79Ang From Ag01            1.93 eA-3
PLAT971_ALERT_2_C Check Calcd Resid. Dens.  1.43Ang From C016            1.87 eA-3
PLAT971_ALERT_2_C Check Calcd Resid. Dens.  1.14Ang From F00U            1.75 eA-3
PLAT971_ALERT_2_C Check Calcd Resid. Dens.  0.94Ang From Ag02            1.64 eA-3
PLAT971_ALERT_2_C Check Calcd Resid. Dens.  1.07Ang From Ag03            1.63 eA-3
PLAT971_ALERT_2_C Check Calcd Resid. Dens.  1.05Ang From Ag03            1.51 eA-3
PLAT972_ALERT_2_C Check Calcd Resid. Dens.  0.83Ang From Ag02           -1.91 eA-3
PLAT972_ALERT_2_C Check Calcd Resid. Dens.  0.89Ang From Ag01           -1.84 eA-3
PLAT972_ALERT_2_C Check Calcd Resid. Dens.  0.84Ang From Ag02           -1.59 eA-3
PLAT977_ALERT_2_C Check Negative Difference Density on H01N      .      -0.46 eA-3
PLAT977_ALERT_2_C Check Negative Difference Density on H01L      .      -0.34 eA-3
```

Alert level G

```
PLAT003_ALERT_2_G Number of Uiso or U(i,j)  Restrained non-H-Atoms         21 Report
PLAT042_ALERT_1_G Calc. and Reported MoietyFormula Strings  Differ     Please Check
              Calc: C29 H10 Ag3 Cl F18 N6 O2
              Rep.: C15 H3 Ag3 F18 N6, C14 H7 Cl O2
PLAT083_ALERT_2_G SHELXL Second Parameter in WGHT  Unusually Large     103.74 Why ?
PLAT116_ALERT_2_G ADDSYM Included (Pseudo) Lattice Translation ...     Please Check
PLAT177_ALERT_4_G The CIF-Embedded .res File Contains DELU Records          2 Report
PLAT178_ALERT_4_G The CIF-Embedded .res File Contains SIMU Records          2 Report
PLAT186_ALERT_4_G The CIF-Embedded .res File Contains ISOR Records          6 Report
PLAT188_ALERT_3_G A Non-default SIMU Restraint Value has been used     0.0200 Report
PLAT188_ALERT_3_G A Non-default SIMU Restraint Value has been used     0.0200 Report
PLAT242_ALERT_2_G Low    'MainMol' Ueq as Compared to Neighbors of       C018 Check
PLAT242_ALERT_2_G Low    'MainMol' Ueq as Compared to Neighbors of       C019 Check
PLAT242_ALERT_2_G Low    'MainMol' Ueq as Compared to Neighbors of       C01C Check
PLAT242_ALERT_2_G Low    'MainMol' Ueq as Compared to Neighbors of       C01F Check
PLAT242_ALERT_2_G Low    'MainMol' Ueq as Compared to Neighbors of       C01I Check
PLAT242_ALERT_2_G Low    'MainMol' Ueq as Compared to Neighbors of       C01M Check
PLAT299_ALERT_4_G Atom Site Occupancy Constrained at ............         0.5 Check
              Cl1     Cl00    H01N    H01L
PLAT301_ALERT_3_G Main Residue  Disorder .............(Resd    1)          2% Note
PLAT434_ALERT_2_G Short Inter HL..HL Contact F00I     ..F00U     .       2.78 Ang.
                                                   x,-1+y,z  =      1_545 Check
PLAT434_ALERT_2_G Short Inter HL..HL Contact F00J     ..F00X     .       2.74 Ang.
                                                   x,-1+y,z  =      1_545 Check
```

```
PLAT605_ALERT_4_G Largest Solvent Accessible VOID in the Structure          9 A**3
PLAT720_ALERT_4_G Number of Unusual/Non-Standard Labels ..........         65 Note
              Ag01    Ag02    Ag03    Cl00    F005    F006    F007    F008
              F009    F00B    F00C    N00D    N00E    N00F    N00G    N00H
              F00I    F00J    N00K    F00L    F00N    O00O    O00P    F00Q
              C00R    H00R    C00S    F00U    F00V    C00W    F00X    C00Y
              C010    C011    C012    H012    C013    C014    C017    H017
              C018    C019    C01A    C01B    H01B    C01C    C01D    H01D
              C01E    C01F    C01G    C01I    C01J    H01J    C01N    H01N
              C01H    C015    C016    H016    C01K    H01K    C01L    H01L
              C01M
PLAT860_ALERT_3_G Number of Least-Squares Restraints .............        228 Note
PLAT868_ALERT_4_G ALERTS Due to the Use of _smtbx_masks Suppressed          ! Info
PLAT883_ALERT_1_G Absent Datum for _atom_sites_solution_primary ..     Please Do !
PLAT912_ALERT_4_G Missing # of FCF Reflections Above STh/L=  0.600        302 Note
PLAT913_ALERT_3_G Missing # of Very Strong Reflections in FCF ....          1 Note
                2  0  4,
PLAT933_ALERT_2_G Number of HKL-OMIT Records in Embedded .res File          1 Note
                6  2 17,
PLAT941_ALERT_3_G Average HKL Measurement Multiplicity ...........        2.3 Low
PLAT969_ALERT_5_G The 'Henn et al.' R-Factor-gap value ...........      4.227 Note
              Predicted wR2: Based on SigI**2  5.31 or SHELX Weight 20.12
PLAT978_ALERT_2_G Number C-C Bonds with Positive Residual Density.          2 Info
```

```
   0 ALERT level A = Most likely a serious problem - resolve or explain
   4 ALERT level B = A potentially serious problem, consider carefully
  24 ALERT level C = Check. Ensure it is not caused by an omission or oversight
  30 ALERT level G = General information/check it is not something unexpected

   2 ALERT type 1 CIF construction/syntax error, inconsistent or missing data
  33 ALERT type 2 Indicator that the structure model may be wrong or deficient
  12 ALERT type 3 Indicator that the structure quality may be low
  10 ALERT type 4 Improvement, methodology, query or suggestion
   1 ALERT type 5 Informative message, check
```

Validation response form

Please find below a validation response form (VRF) that can be filled in and pasted into your CIF.

```
# start Validation Reply Form
_vrf_PLAT213_lcz071
;
PROBLEM: Atom F00X            has ADP max/min Ratio .....        3.1 prolat
RESPONSE: ...
;
_vrf_PLAT234_lcz071
;
PROBLEM: Large Hirshfeld Difference Ag01     --N00E     .       0.17 Ang.
RESPONSE: ...
;
_vrf_PLAT241_lcz071
;
PROBLEM: High   'MainMol' Ueq as Compared to Neighbors of       O00P Check
RESPONSE: ...
;
```

```
_vrf_PLAT242_lcz071
;
PROBLEM: Low    'MainMol' Ueq as Compared to Neighbors of       Ag01 Check
RESPONSE: ...
;
_vrf_PLAT906_lcz071
;
PROBLEM: Large K Value in the Analysis of Variance ......     19.946 Check
RESPONSE: ...
;
_vrf_PLAT911_lcz071
;
PROBLEM: Missing FCF Refl Between Thmin & STh/L=    0.600         47 Report
RESPONSE: ...
;
_vrf_PLAT934_lcz071
;
PROBLEM: Number of (Iobs-Icalc)/Sigma(W) > 10 Outliers ..          1 Check
RESPONSE: ...
;
_vrf_PLAT971_lcz071
;
PROBLEM: Check Calcd Resid. Dens.  0.81Ang From Ag02            2.14 eA-3
RESPONSE: ...
;
_vrf_PLAT972_lcz071
;
PROBLEM: Check Calcd Resid. Dens.  0.83Ang From Ag02           -1.91 eA-3
RESPONSE: ...
;
_vrf_PLAT977_lcz071
;
PROBLEM: Check Negative Difference Density on H01N      .      -0.46 eA-3
RESPONSE: ...
;
# end Validation Reply Form
```

It is advisable to attempt to resolve as many as possible of the alerts in all categories. Often the minor alerts point to easily fixed oversights, errors and omissions in your CIF or refinement strategy, so attention to these fine details can be worthwhile. It is up to the individual to critically assess their own results and, if necessary, seek expert advice.

PLATON version of 17/09/2025; check.def file version of 17/09/2025

---

duplicate check

No duplication found

Datablock lcz071 - ellipsoid plot

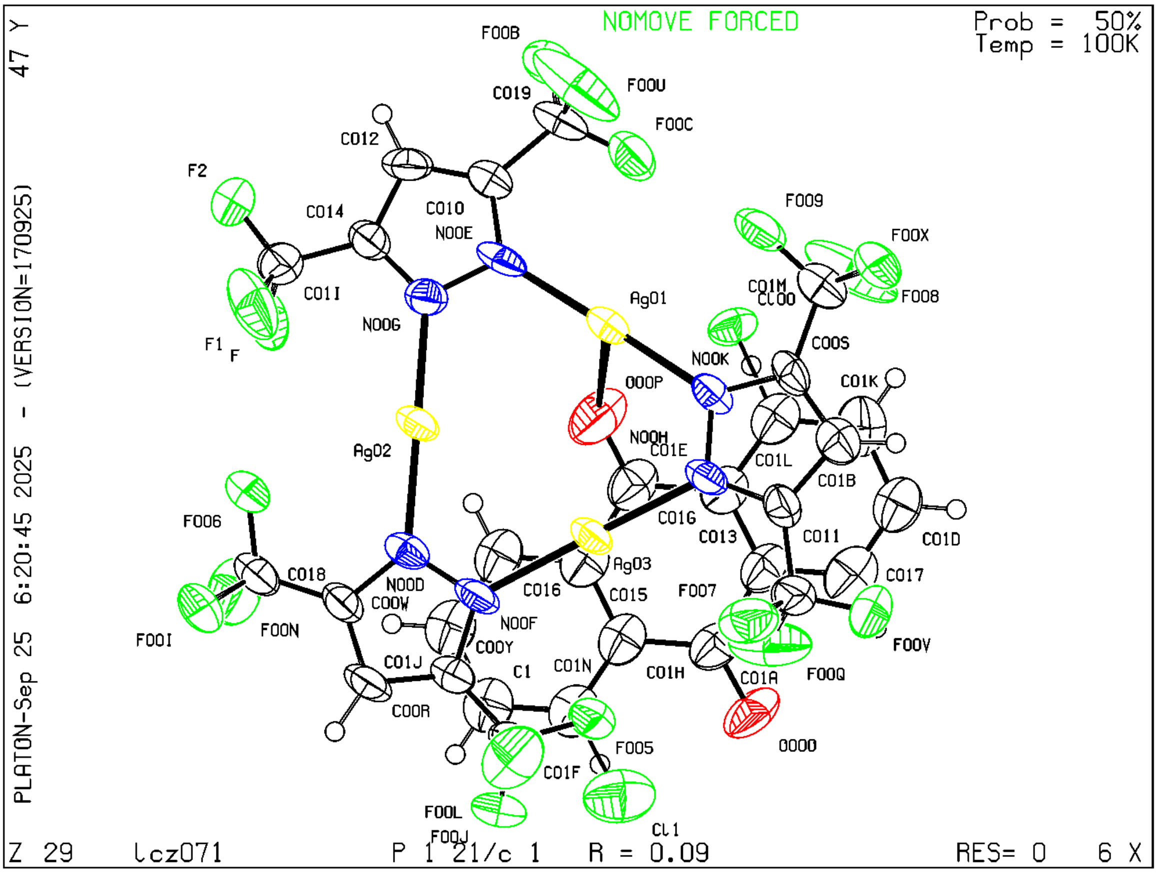

Supplement: Supplementary file 2 — Supporting File 2: anie72462‐sup‐0002‐DataFile.zip. [file ANIE-65-e1218503-s002.zip › supplementary_materials_crystallographic_data submit.zip!/cif_and_checkcif(1-114)/Ag3Pz3_71/Ag3Pz3_71_checkcif.pdf]
